# Quality assurance of hematopoietic stem cells by macrophages determines stem cell clonality*

**DOI:** 10.1126/science.abo4837

**Published:** 2022-09-22

**Authors:** Samuel J. Wattrus, Mackenzie L. Smith, Cecilia Pessoa Rodrigues, Elliott J. Hagedorn, Ji Wook Kim, Bogdan Budnik, Leonard I. Zon

**Affiliations:** 1 Stem Cell Program and Division of Hematology/Oncology, Boston Children’s Hospital and Dana Farber Cancer Institute, Howard Hughes Medical Institute, Harvard Medical School; Boston MA, USA; 2 Harvard Stem Cell Institute, Stem Cell and Regenerative Biology Department, Harvard University; Cambridge, MA; 3 Mass Spectrometry and Proteomics Resource Laboratory, Faculty of Arts and Sciences Division of Science, Harvard University, Cambridge MA, USA

## Abstract

Tissue-specific stem cells persist for a lifetime and can differentiate to maintain homeostasis or transform to initiate cancer. Despite their importance, there are no described quality assurance mechanisms for newly formed stem cells. We observed intimate and specific interactions between macrophages and nascent blood stem cells in zebrafish embryos. Macrophage interactions frequently led to either removal of cytoplasmic material and stem cell division, or complete engulfment and stem cell death. Stressed stem cells were marked by surface Calreticulin, which stimulated macrophage interactions. Using cellular barcoding, we found that calreticulin knock-down or embryonic macrophage depletion reduced the number of stem cell clones that established adult hematopoiesis. Our work supports a model in which embryonic macrophages determine hematopoietic clonality by monitoring stem cell quality.

## One-Sentence Summary

Macrophages selectively expand or eliminate individual stem cells during development on the basis of surface Calreticulin.

Tissue stem cells born during embryogenesis support homeostasis for life. Despite the importance of these cells for proper tissue function, there are no described quality assurance mechanisms for newly formed stem cells. To explore this possibility, we studied zebrafish embryonic blood development. Hematopoietic stem and progenitor cells (HSPCs) emerge from the ventral wall of the dorsal aorta (VDA), enter circulation, and lodge in the embryonic niche - a vascular plexus called the caudal hematopoietic tissue (CHT) (1, 2). HSPCs rapidly expand in the CHT for 3 to 4 days before migrating to the kidney marrow, the adult hematopoietic niche. In both niches, HSPCs interact with a variety of cell types, including vascular endothelial cells, mesenchymal stromal cells, and macrophages (MΦs) (3–6). *In vivo* clonal labeling shows that 20 – 30 of the hematopoietic stem cell (HSC) clones born in the VDA ultimately give rise to the adult blood system (7). It remains unclear if nascent HSCs from the VDA undergo quality assurance before establishing adult hematopoiesis. Here, using live imaging and cellular barcoding, we found discrete interactions between stem cells and embryonic macrophages that regulated the number of long-lived hematopoietic stem cells clones that produce blood in adulthood.

## Results:

### Macrophages interact with nascent HSPCs in the CHT

**Fig. 1.**
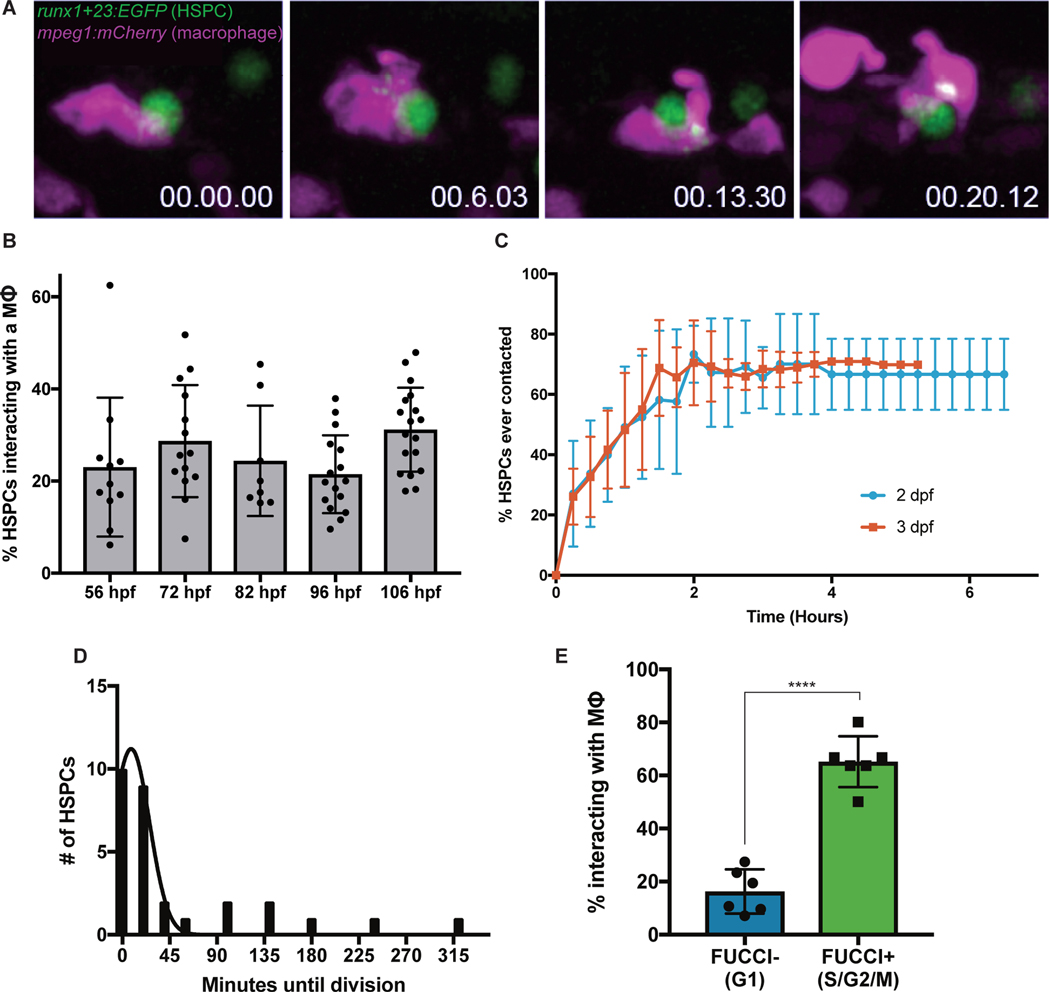
Macrophages make intimate interactions with newly formed HSPCs. (**A**) Time lapse live-imaging identifies prolonged cell-cell contacts between *runx1+23:EGFP*^+^ HSPCs and *mpeg1:mCherry*^+^ primitive macrophages involving exchange of fluorescent material. (**B**) Approximately 20–30% of HSPCs interact with macrophages in the CHT at any one time from 56 hpf to 106 hpf. Mean +/− s.d. (**C**) High-resolution tracking of individual *runx1+23:mCherry*^*+*^ cells over several hours in the CHT reveals that the majority of HSPCs eventually make sustained contact with macrophages (> 5 minutes). Mean +/− s.d. (**D**) HSPCs frequently complete a cell division shortly after macrophage interactions. Approximately 81% of HSPC divisions occur within 30 minutes of a macrophage interaction. Mean +/− s.d. (**E**) Around 65% of Fucci^+^ HSPCs in S/G2/M phases interact with macrophages at any one time, as compared to less than 20% of Fucci^−^ HSPCs. Mean +/− s.d., Unpaired t test; ****P<0.0001.

Macrophages help maintain homeostasis by modulating inflammation, producing cytokines, and patrolling to clear dead, stressed, or aged cells (8–10). Given these roles in somatic tissue, the enrichment of macrophages in the CHT, and previous observations between macrophages and hematopoietic cells (6), we investigated macrophage function in the niche. We undertook high-resolution live imaging using *mpeg1:mCherry;runx1+23:EGFP* zebrafish embryos with mCherry^+^ macrophages and EGFP^+^ HSPCs (4, 11). Shortly after lodgment in the CHT, HSPCs were contacted by a nearby macrophage and their surfaces were scanned. These interactions sometimes resulted in the uptake of fluorescent HSPC material by the macrophage (Fig. 1A; Movie S1). From 56 to 106 hours post-fertilization (hpf), approximately 20–30% of HSPCs were engaged by a macrophage at any timepoint (Fig. 1B). These interactions were specific to HSPCs; macrophage engagement with erythrocytes and endothelial cells was significantly lower (0.6 – 3.9% of erythrocytes and 0.5 – 6.7% of endothelial cells) (Fig. S1A). Macrophages contacted HSPCs for up to 45 minutes - sometimes taking up fluorescent HSPC material. We classified interactions into three types: prolonged cell-cell contact, “grooming” during which the HSPC was left intact but had a small portion of cellular material taken up by the macrophage, or “dooming” during which the HSPC was fully engulfed and destroyed by the macrophage (Movie S2). We also found similar interactions with other HSPC-reporters *cd41:GFP* and *cmyb:GFP* (Figs. S1D–E). To examine if macrophage-HSPC interactions occurred in mammals, we studied E14.5 murine fetal liver sections by immunofluorescence and found that 33% of c-Kit^+^ hematopoietic cells were in contact with F4/80^+^ macrophages. This included c-Kit^+^ cells being pinched or fully engulfed by macrophages, similar to our observations in zebrafish (Fig. S1F). Overall, these data identify novel macrophage-HSPC interactions in the embryonic hematopoietic niche.

To better characterize macrophage-HSPC interactions, we tracked individual HSPCs in the CHT at 2- or 3-days post-fertilization (dpf) and recorded macrophage interactions. We found that 70% of HSPCs experienced prolonged macrophage contact over a 3-hour imaging period (Fig. 1C). Within this timeframe, 13% of these HSPCs were groomed and 13% were doomed. Some HSPCs were contacted by macrophages multiple times and underwent grooming or dooming after repeated interaction, suggesting that the majority of HSPCs may eventually undergo grooming or dooming at some point during the 3–4 days that they occupy the CHT. Eighty-one percent of HSPC divisions occurred within 30 minutes of grooming or prolonged contact (Fig. 1D). Using the *Tg(EF1a:mAG-zGem(1/100))^rw0410h^* (Fucci) transgene (12) labeling cells in S/G2/M phases of the cell cycle, we found that approximately 65% of Fucci^+^ HSPCs contacted macrophages, compared to only 16% of Fucci^−^ HSPCs (Fig. 1E). We next assessed the viability of HSPCs engulfed by macrophages. Staining for cell death with Acridine Orange or an Annexin V-YFP construct (13) showed almost no apoptotic HSPCs in the CHT that were not already engulfed by macrophages (Figs. S1B–C). Only after full engulfment did HSPCs exhibit apoptosis (Movie S3). Together these data identify a set of macrophage-HSPC interactions that either precede HSPC division or death.

### A subset of primitive macrophages regulates stem cell clone number

**Fig. 2.**
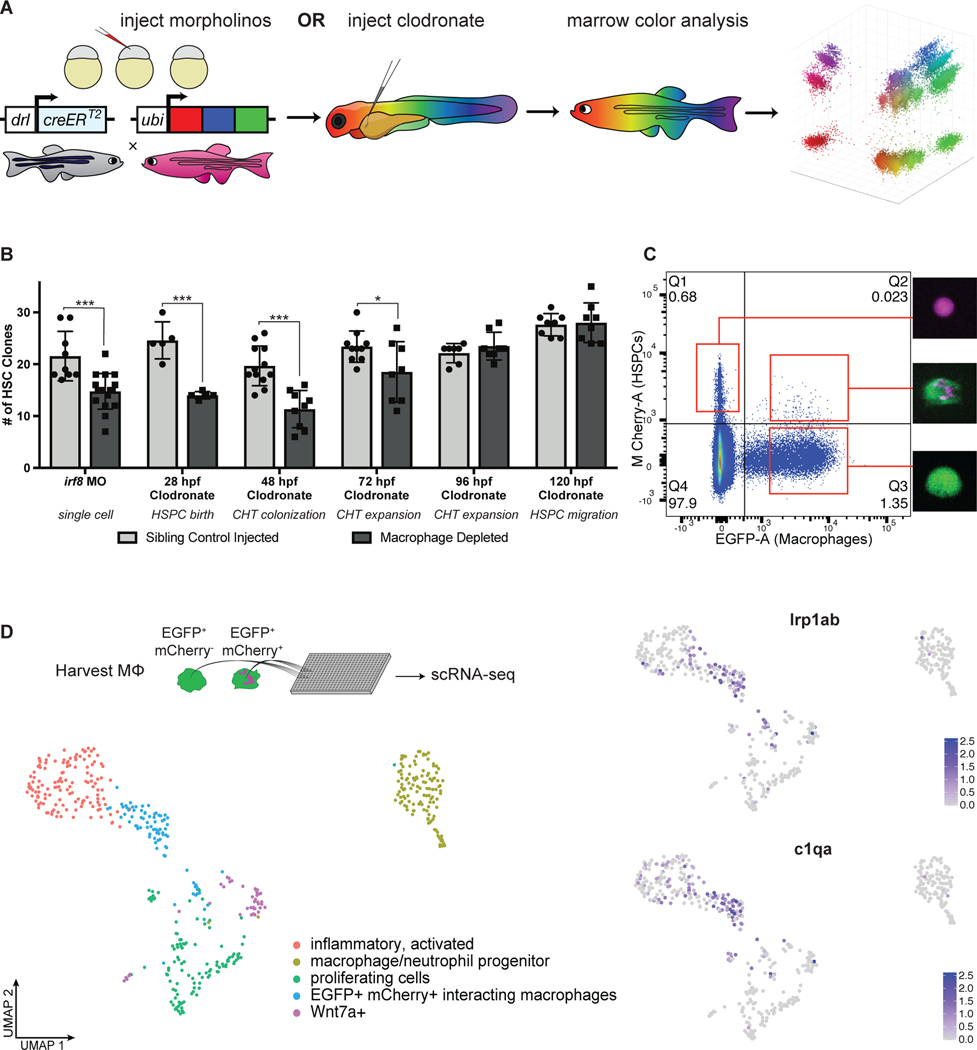
Macrophages in the CHT regulate stem cell clonality. (**A**) A schematic overview of the *Zebrabow-M* system: animals with 15–20 insertions of a multicolor fluorescent cassette are crossed to the *draculin:CreER*^*T2*^ line to enable clonal labeling of lateral plate mesoderm lineages. By treating with 4-OHT at 24 hpf just after HSC specification, individual stem cell lineages express unique fluorescent hues which can be quantified in the adult marrow. (**B**) Families of *Zebrabow-M;draculin:CreER*^*T2*^ animals injected with either clodronate liposomes or the *irf8* morpholino exhibit reduced numbers of HSC clones in the adult marrow, even when macrophages are not depleted until after emergence from the VDA. Mean +/− s.d., Unpaired t test; *P<0.05, ***P<0.001. (**C**) Macrophages (*mpeg1:EGFP*^+^) which have interacted with HSPCs (*runx1+23:mCherry*^+^) and removed fluorescent material can be harvested by FACS. (**D**) Macrophages which engage HSPCs are marked by *lrp1ab* and *c1qa*. Spectral scale reports z-scores.

As we saw proliferation following macrophage-HSPC interactions, we next sought to determine if this might influence the number of stem cell clones contributing to adult hematopoiesis. We used TWISTR (tissue editing with inducible stem cell tagging via recombination) (14) to combine morpholino mediated gene knock-down with Zebrabow HSC color labeling. *Zebrabow-M;draculin:CreER^T2^* embryos enable unique lineage labeling of individual HSC clones at 24 hpf (Fig. 2A) (7, 15). To deplete embryonic macrophages, we injected the *irf8* morpholino to block macrophage formation (16) or delivered clodronate liposomes to ablate macrophages at various timepoints: 28 hpf, before HSPC emergence in the VDA, 48 hpf, before HSPC lodgment in the CHT, 72 hpf, after HSPC lodgment in the CHT, 96 hpf, after HSPCs have doubled (4), or 120 hpf, as HSPCs start to colonize the marrow. Zebrabow analysis of adult marrow myelomonocytes revealed a consistent reduction in hematopoietic clonality compared to sibling controls when macrophages were depleted before 96 hpf (Fig. 2B). These results demonstrate that embryonic macrophages regulate HSC clone number after VDA emergence and niche colonization until at least one round of amplification has completed.

To better understand the mechanism and cellular consequences of macrophage-HSPC interactions, we pursued transcriptomic analysis of niche macrophages. Because macrophages can take up fluorescent material from HSPCs, we reasoned it would be possible to identify interacting macrophages by their fluorescence profile. Indeed, flow cytometry of dissociated *mpeg1:EGFP;runx1+23:mCherry* embryos revealed a rare population of EGFP^+^mCherry^+^ cells morphologically consistent with macrophages containing HSPC fragments (Fig. 2C). We dissected embryonic zebrafish tails and purified interacting macrophages (EGFP^+^mCherry^+^) and non-interacting macrophages (EGFP^+^mCherry^−^) for single-cell mRNA sequencing. We identified a single population of macrophages which segregated by both gene expression and mCherry fluorescence (Fig. 2D). These cells were enriched for genes associated with engulfment, lysosomal degradation, and cholesterol transport and were marked by genes including *hmox1a, ctsl.1, slc40a1, lrp1ab,* and *c1qa* (Fig. 2D; Fig. S2A). We validated these data with a fluorescent cholesterol mimic, Lysotracker dye, and *in situ* hybridization (Figs. S2B–D). Together, these data show that a transcriptionally distinct and relatively homogenous subset of macrophages engage HSPCs in the CHT.

### Surface Calreticulin drives macrophage-HSPC interactions

**Fig. 3.**
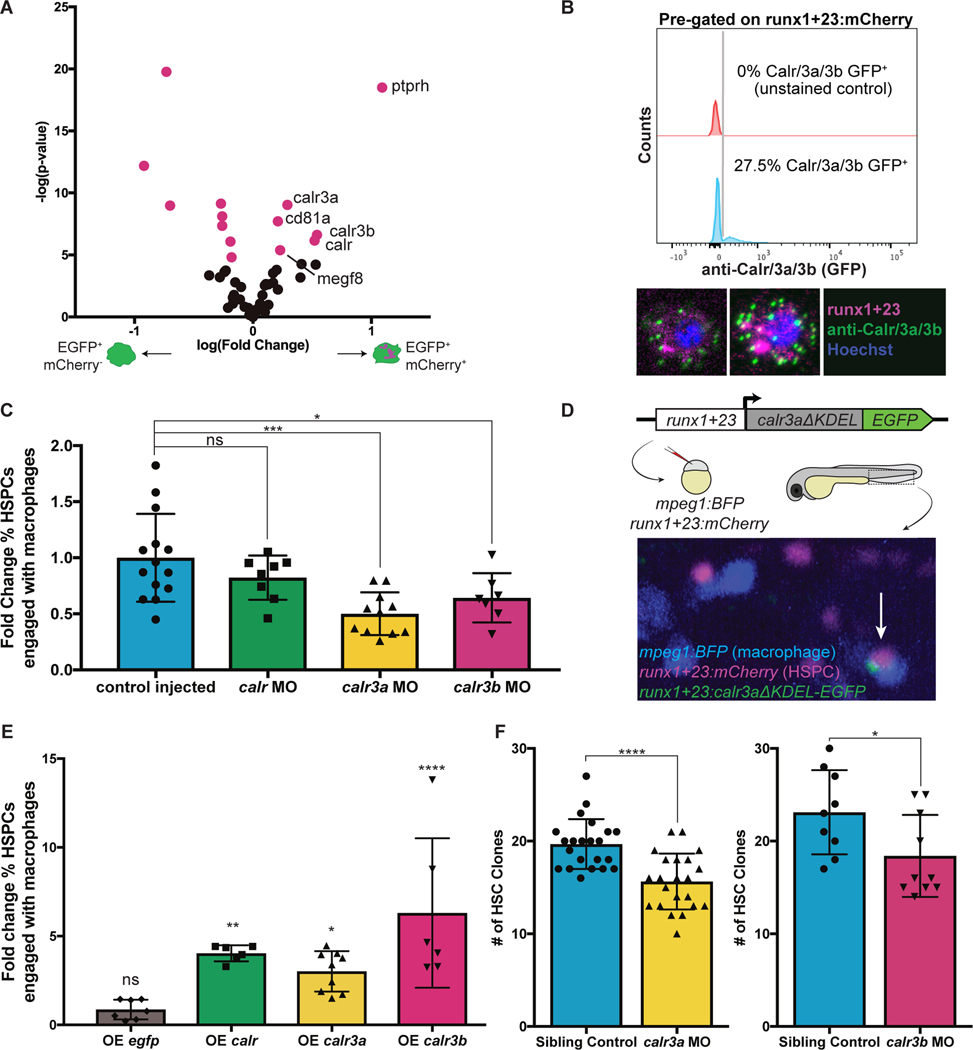
Calreticulin drives HSPC-macrophage interactions to regulate clonality. (**A**) Analysis of differentially enriched potential surface proteins from interacting macrophages identifies three paralogs of Calreticulin. (**B**) Flow cytometry shows ~30% of *runx1+23:mCherry*^+^ HSPCs stain for surface Calreticulin. (**C**) Morpholino knock-down of *calr3a* or *calr3b* significantly reduces the fraction of HSPCs interacting with macrophages at any one time. Mean +/− s.d., One-way ANOVA with Dunnett’s multiple comparisons test; *P<0.05, ***P<0.001. (**D**) Calreticulin paralogs without the ER-retention KDEL sequence were fused to EGFP, driven by the HSPC-specific *runx1+23* enhancer, and injected into stable *runx1+23:mCherry;mpeg1:BFP* embryos. Mosaic animals overexpress Calreticulin in a random subset of HSPCs. Arrow indicates an HSPC overexpressing *calr3a* engaged by a macrophage. (**E**) HSPCs overexpressing *calr, calr3a*, or *calr3b* are more frequently engaged by macrophages compared to non-overexpressing HSPCs in the same embryos. Overexpressing *egfp* alone has no effect. Mean +/− s.d., One-way ANOVA with Dunnett’s multiple comparisons test; *P<0.05, **P<0.01, ****P<0.0001. (**F**) Knock-down of *calr3a* or *calr3b* reduces the number of HSC clones that contribute to adult hematopoiesis. Mean +/− s.d., Unpaired t test; *P<0.05, ****P<0.0001.

To gain insight into the proteinaceous material taken up by macrophages, we pursued a modified form of single-cell proteomics called few-cell proteomics (17) to compare interacting to non-interacting macrophages. We identified 203 peptides enriched in interacting macrophages, potentially representing a repertoire of proteins either involved in the process of macrophage-HSPC interaction or taken directly from HSPCs. To identify molecular patterns recognized on HSPCs, we excluded peptides with enriched transcripts in interacting macrophages and compared the remaining peptides to the Cell Surface Protein Atlas (18). Notably, surface peptides enriched in interacting macrophages included three Calreticulin paralogs: *calr, calr3a,* and *calr3b* (Fig. 3A). Though Calreticulin is widely expressed and typically functions as a chaperone protein in the endoplasmic reticulum, it can also sometimes be displayed on the cell surface as an “eat-me” signal (9, 10, 19). Based on our proteomic results, we hypothesized that HSPCs could display surface Calreticulin, stimulating macrophage interactions. We found that 30% of HSPCs at 72 hpf exhibited classic punctate surface Calreticulin staining (20) (Fig. 3B), similar to the percentage of HSPCs interacting with macrophages *in vivo* (Fig. 1B). Additionally, the canonical surface Calreticulin binding partners, *lrp1ab* and *c1qa,* were transcriptionally enriched in interacting macrophages (Fig. 2D). Together, Lrp1ab and C1qa contact Calreticulin and form a bridging complex to initiate phagocytic activity (10, 20, 21). These results show that Calreticulin decorates the surface of HSPCs and may promote macrophage interaction.

To study the role of Calreticulin in macrophage-HSPC interactions, we used morpholinos to knock-down Calreticulin gene expression. Knock-down of *calr3a* or *calr3b* significantly reduced the percentage of HSPCs engaged by macrophages (Fig. 3C). This effect was reversed in genetic rescue experiments (Figs. S3A–B). We then generated parabiotic fusions of embryos with or without Calreticulin knock-down and found that knock-down HSPCs had reduced interactions with control macrophages. In contrast, control HSPCs had normal levels of interaction with Calreticulin knock-down macrophages, indicating that Calreticulin presentation is HSPC-autonomous (Figs. S3C–D). Next, we tested the effect of constitutively surface-translocated Calreticulin expressed under the HSPC-specific *runx1+23* enhancer (4) (Fig. 3D). Injecting this construct to early embryos generated mosaic animals, which permitted direct comparison of HSPCs with or without Calreticulin overexpression. HSPCs overexpressing *calr, calr3a,* or *calr3b* were 3 to 5-fold more likely to interact with macrophages compared to non-overexpressing HSPCs in the same embryo (Fig. 3E). When *calr3a* or *calr3b* were knocked down, prolonged contact, grooming, and dooming interactions all decreased, with a more severe decrease in dooming (Fig. S3E). In contrast, nearly all cells overexpressing Calreticulin were doomed (Fig. S3F). Taken together, these data show that surface Calreticulin promotes macrophage-HSPC interactions and suggest that differing levels of Calreticulin determine whether an HSPC experiences prolonged contact, is groomed, or is doomed.

To determine if Calreticulin-dependent interactions during development were responsible for regulating HSC clonality into adulthood, we knocked down *calr3a* or *calr3b* and color labeled HSCs at 24 hpf. Adult Zebrabow analysis of morphants showed a significant reduction in the number of HSC clones compared to sibling controls (Fig. 3F). These data show that Calreticulin-dependent interactions in development support a greater number of long-lived HSC clones.

### Macrophages buffer HSPC stress and promote divisions

**Fig. 4.**
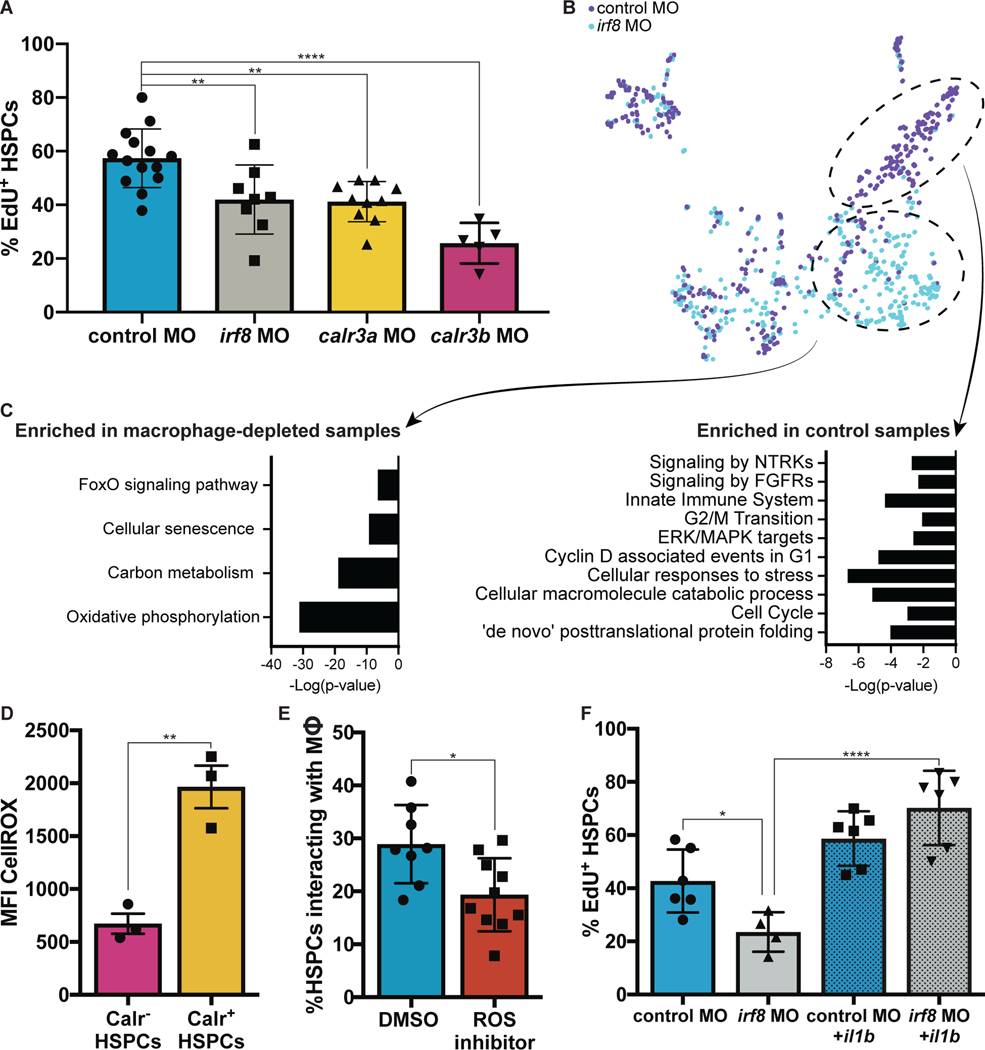
Macrophages buffer HSPC stress and regulate HSPC expansion. (**A**) EdU staining of *runx1+23:mCherry* embryos injected with either the *calr3a*, *calr3b*, or *irf8* morpholinos identifies significant reduction in proliferating HSPCs at 3 dpf. Mean +/− s.d., One-way ANOVA with Dunnett’s multiple comparisons test; **P<0.01, ****P<0.0001. (**B**)(**C**) Single-cell mRNA-seq analysis of *runx1+23*^+^ FACS-purified cells from *irf8* or control morphants reveals a population of stressed HSPCs that persist in the absence of macrophages and a population of cycling cells enriched in the control sample. (**D**) Embryonic HSPCs marked by surface Calreticulin exhibit higher levels of ROS. (**E**) ROS inhibition with diphenylene iodonium significantly reduces macrophage-HSPC interactions. Mean +/− s.d., Unpaired t test; *P<0.05. (**F**) Expression of *il1b* by heat shock rescues the effect of macrophage depletion on HSPC proliferation. Mean +/− s.d., One-way ANOVA with Sidak’s multiple comparisons test; *P<0.05, ****P<0.001.

We next assessed Calreticulin function in HSPC development. To analyze changes to HSPC emergence, we injected the *irf8, calr3a,* or *calr3b* morpholino into *cd41:GFP;kdrl:mCherry* embryos to visualize the endothelial-to-hematopoietic transition (22). Quantification of EGFP^+^mCherry^+^ cells in the VDA revealed no significant difference in HSPC budding (Fig. S4A). Serial imaging of *cd41:GFP^+^* cells over early development revealed that knock-down of *calr3a* or *calr3b* did not affect HSPC numbers through 60 hpf, but later reduced HSPCs at 72 and 84 hpf (Figs. S4B–C). This was not due to apoptosis or altered trafficking to the kidney marrow (Figs. S4D–E). Rather, depletion of macrophages or knock-down of *calr3a* or *calr3b* significantly reduced the fraction of proliferative HSPCs in the CHT at 72 hpf, as measured by EdU incorporation (Fig. 4A). This corroborates the association of macrophage-HSPC interactions with HSPC division identified by live imaging (Figs. 1D–E). These data show that Calreticulin-dependent macrophage-HSPC interactions serve to expand and maintain HSPCs during early development by promoting proliferation in the CHT.

To molecularly evaluate the effect of macrophage interactions on HSPCs and the qualities that lead to surface Calreticulin, we injected *runx1+23:mCherry* embryos with the *irf8* or control morpholino and performed single-cell mRNA-seq on HSPCs at 72 hpf. This analysis identified a population of HSPCs enriched in *irf8* morphants marked by genes associated with FoxO activity and cellular senescence (Figs. 4B–C). FoxO activity initiates in response to elevated reactive oxygen species (ROS) and mediates detoxification of ROS and repair of ROS-induced damage (23, 24). In murine HSCs, FoxO deletion and ROS accumulation results in dysregulation of apoptosis, cell cycling, and colony formation (25). The enrichment of HSPCs with FoxO activity in *irf8* morphants suggested potential ROS accumulation which is ordinarily resolved by macrophages. In agreement with this, flow cytometric analysis showed higher ROS levels in HSPCs marked by surface Calreticulin, with significant correlation between ROS levels and surface Calreticulin intensity (Spearman’s Correlation; ****P< 2.2e-16) (Fig. 4D). Inhibiting ROS with diphenylene iodonium reduced macrophage-HSPC interactions (Fig. 4E), whereas elevating ROS with hydrogen peroxide or D-Glucose (26) increased macrophage-HSPC interactions (Fig. S5A). Consistent with prior work linking ROS, ER stress, and surface Calreticulin (27), ER stress inhibition by *perk* knock-down also decreased macrophage-HSPC interactions (Fig. S5B). These data show that without macrophages, a population of HSPCs with elevated ROS accumulates, and that higher ROS levels correlate with surface Calreticulin.

Embryos injected with the *irf8* morpholino also had fewer HSPCs marked by genes associated with cell cycling and ERK/MAPK signaling (Figs. 4B–C). In accordance with this, depleting macrophages or inhibiting ERK/MAPK without reducing macrophage interactions decreased HSPC proliferation. ERK/MAPK inhibition in the context of macrophage depletion did not further reduce proliferation, indicating that macrophages likely stimulate division via this pathway (Fig. S5C). As inflammation is a critical developmental determinant of HSPC proliferation (28, 29), we reasoned that cytokines expressed by the macrophages, such as *il1b,* could be responsible for HSPC divisions (Fig. S5D) (29, 30). To investigate this possibility, we generated parabiotic fusions of control-injected embryos to *il1b* morpholino-injected embryos (31) and evaluated interactions between HSPCs and macrophages from both parabionts (Fig. S5E). Interactions with control macrophages led to significantly more HSPC divisions than interactions with *il1b* knock-down macrophages, showing that macrophage-produced Il1b promotes HSPC division (Fig. S5F). Additionally, heat shock overexpression of *il1b* rescued HSPC proliferation after macrophage depletion (29) (Fig. 4F). ERK/MAPK inhibition abolished the effect of *il1b* (Fig. S5G), indicating that Il1b*-*mediated HSPC divisions act through ERK/MAPK, matching the proliferation signature identified by single-cell mRNA-sequencing (Figs. 4B–C). These results show that HSPC cycling in the CHT is mediated through ERK/MAPK activity induced by macrophage-derived Il1b.

Our data support a model in which macrophages of the embryonic niche vet the quality of newly formed HSPCs through prolonged physical contact leading to either expansion or engulfment. This process is mediated by display of cell surface Calreticulin, which is associated with elevated ROS. It has previously been reported that metabolic shifts during HSPC generation in the VDA elevate ROS to mediate HIF1α stabilization (26). Cells with high ROS are also at elevated risk for DNA damage and dysfunction (23, 24). Our work suggests that while ROS promotes stem cell emergence in the VDA, titration of ROS is ultimately required for normal hematopoiesis. Although we see no evidence for *vcam1* expression in embryonic macrophages, previous studies have indicated a role for macrophages in HSPC homing (6). Macrophages are involved in HSPC mobilization in the VDA (32), and murine macrophage subpopulations facilitate HSPC engraftment (33). In contrast, our studies find that macrophages in the CHT remove clones with high surface Calreticulin which have not downregulated ROS. Healthy HSPCs with low-to-moderate ROS and Calreticulin experience prolonged macrophage contact and grooming, avoid complete engulfment, and respond to pro-proliferative Il1b enabling competition for marrow colonization.

Our work establishes that stem cells are quality assured for stress levels during development, and this impacts the clones that contribute to blood formation in adulthood. Calreticulin functions as an “eat-me” molecule that initiates macrophage-HSPC interaction and leads to programmed cell clearance or stem cell expansion. Orthologs of CD47 and SIRPα, “don’t-eat-me” signals, have not been identified in zebrafish, but other primitive signals could influence macrophage behaviors. This quality assurance mechanism may also operate in adulthood in response to environmental stress, such as during marrow transplantation or in clonal stem cell disorders including myelodysplasia and leukemia. Macrophages may selectively expand or remove clones of tissue-specific stem cells in other systems similar to our findings. Other tissue stem cells rely on macrophages to assure adequate tissue regeneration (34), which could occur through selective proliferation of certain clones. Manipulating this quality assurance mechanism may have significant therapeutic implications for stem cell disorders and tissue regeneration.

## Supplementary Material

Video S1

Video S2

Video S3

Supp Fig 1

Supp Fig 2

Supp Fig 3

Supp Fig 4

Supp Fig 5

Table S1

Table S2

Supplement

## Data Availability

All data are available in the main text or the supplementary materials. The scRNA-seq data are available in the NCBI Gene Expression Omnibus (GSE196553). Proteomic data are available in the MassIVE database (MSV000088780) and PRIDE repository (PXD031434).
