# Supplementary figures and images for: Quality assurance of hematopoietic stem cells by macrophages determines stem cell clonality
Source: Science. Author manuscript; Available in PMC 2022 Sep 30. (PMC9524573; doi:10.1126/science.abo4837)

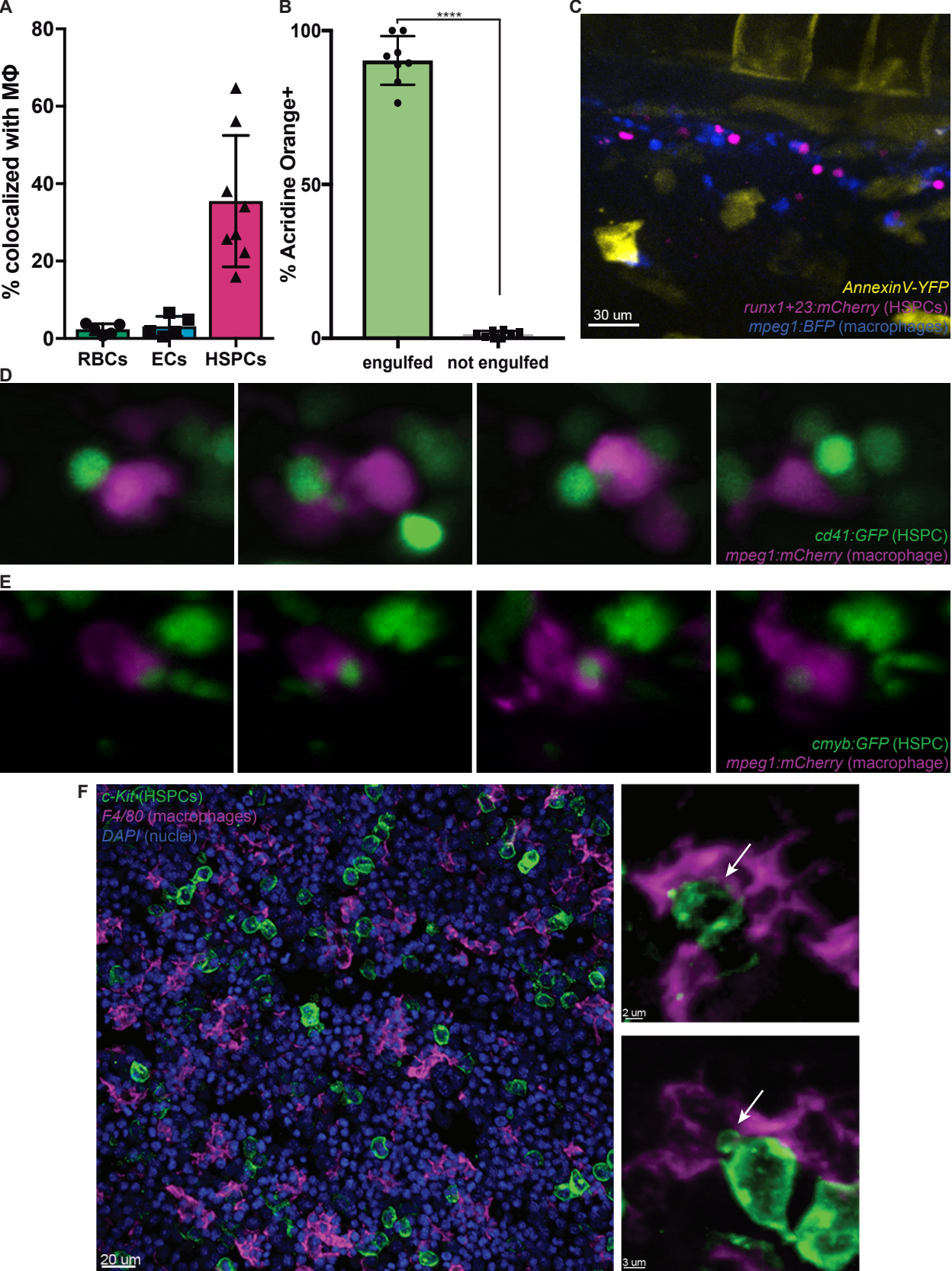

Supplemental Figure 1

Supplement: Supp Fig 1 [file NIHMS1835312-supplement-Supp_Fig_1.pdf]

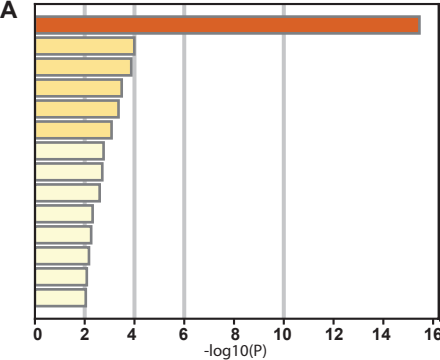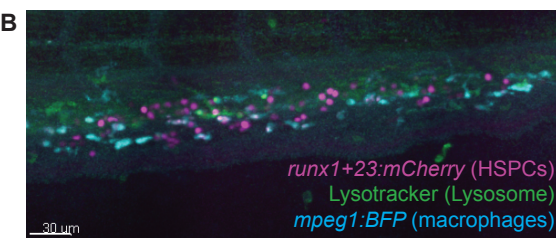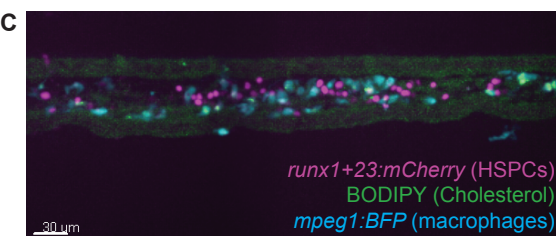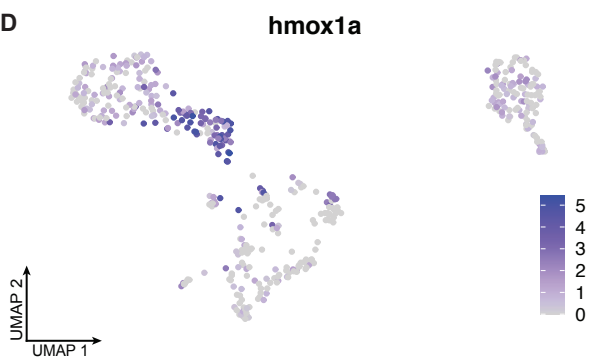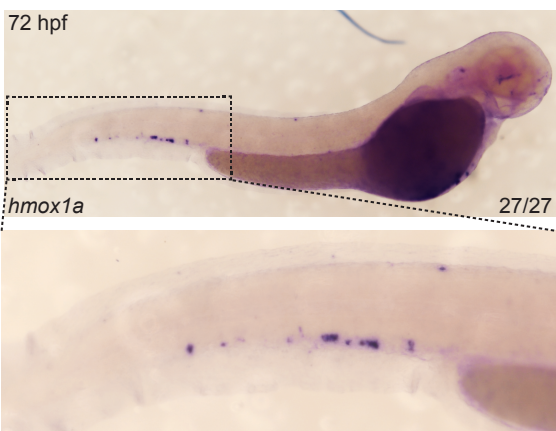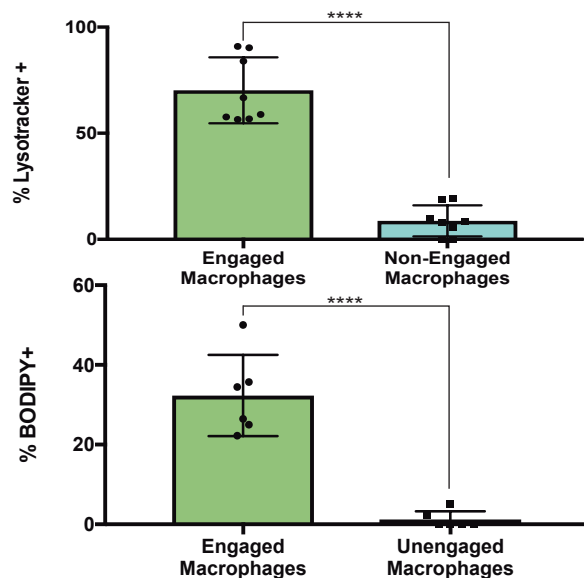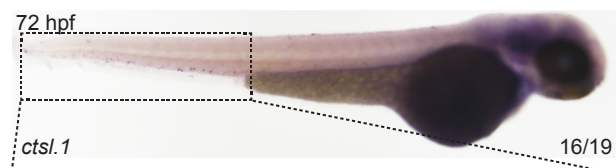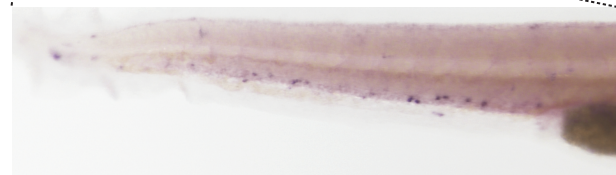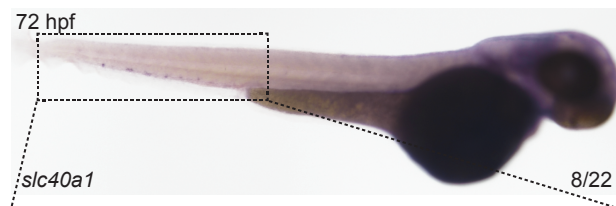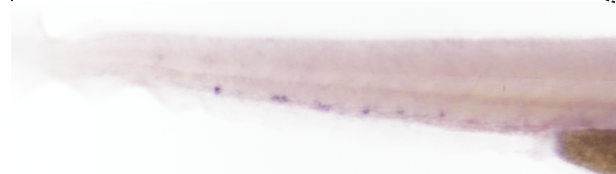

**Supplemental Figure 2**

Supplement: Supp Fig 2 [file NIHMS1835312-supplement-Supp_Fig_2.pdf]

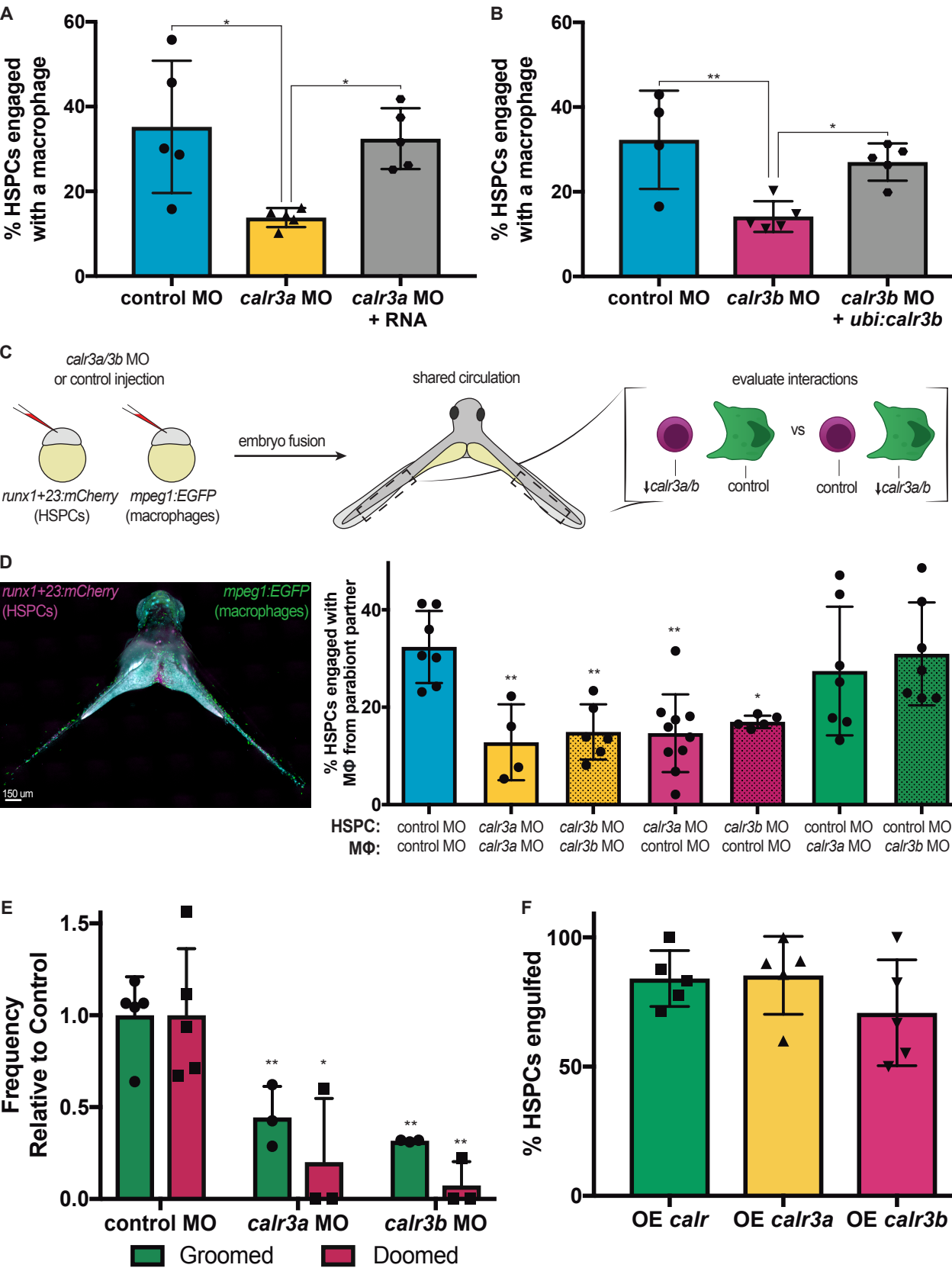

Supplemental Figure 3

Supplement: Supp Fig 3 [file NIHMS1835312-supplement-Supp_Fig_3.pdf]

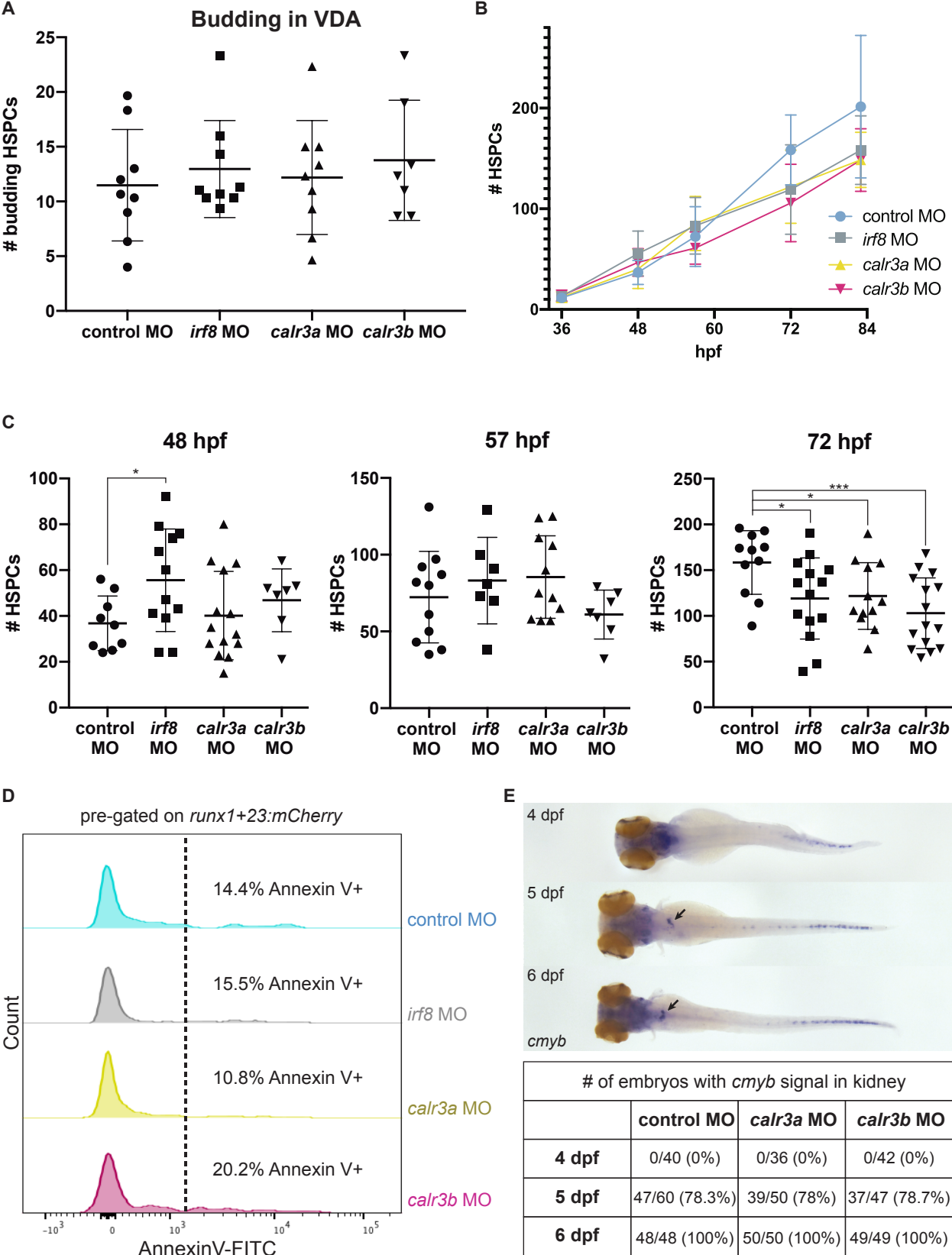

**Supplemental Figure 4**

Supplement: Supp Fig 4 [file NIHMS1835312-supplement-Supp_Fig_4.pdf]

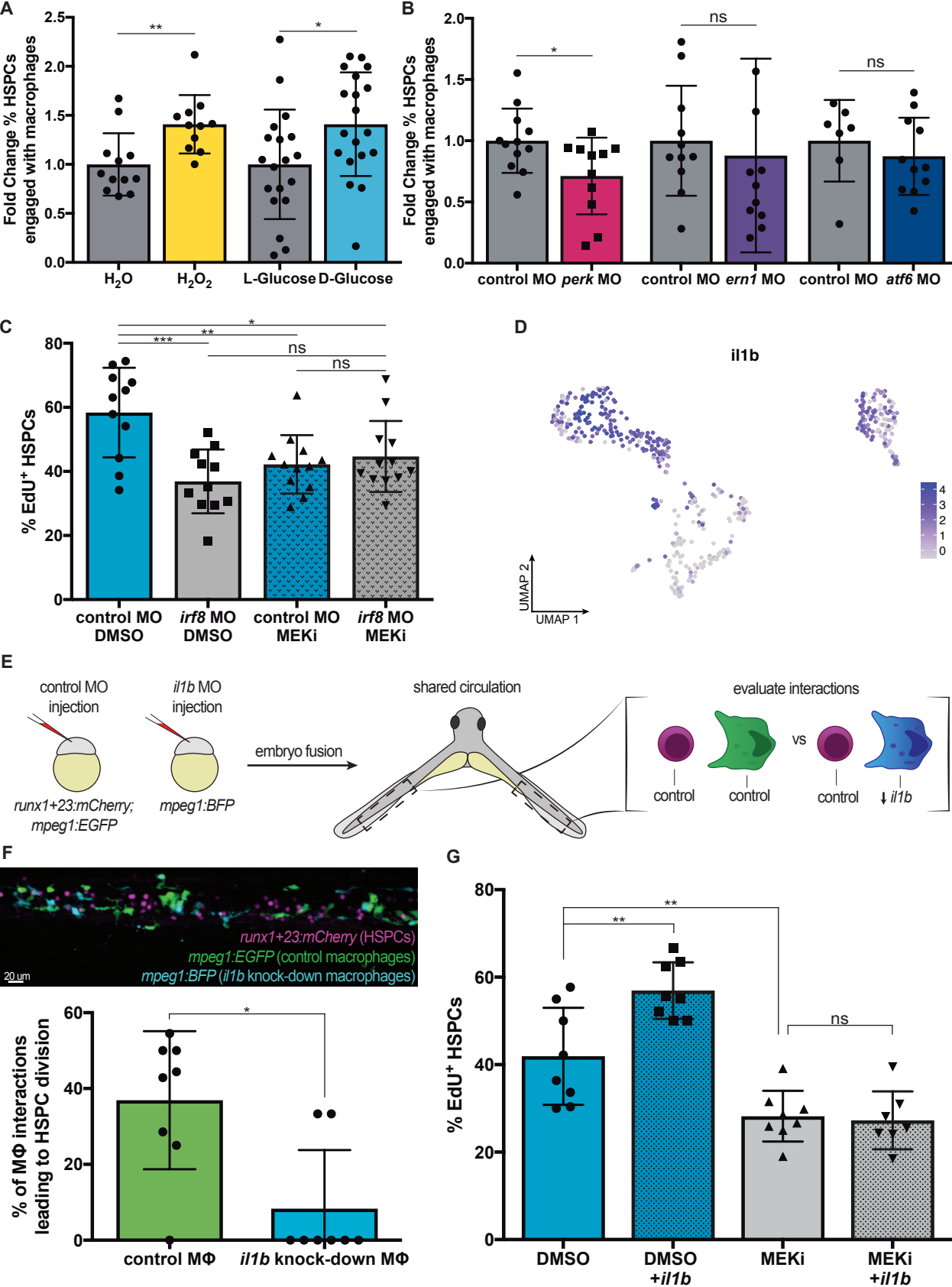

Supplemental Figure 5

Supplement: Supp Fig 5 [file NIHMS1835312-supplement-Supp_Fig_5.pdf]
