# Supplementary material for: Quality assurance of hematopoietic stem cells by macrophages determines stem cell clonality
Source: Science. Author manuscript; Available in PMC 2022 Sep 30. (PMC9524573; doi:10.1126/science.abo4837)
Supplement: Table S1 [file NIHMS1835312-supplement-Table_S1.pdf]

Table S1. Primer sequences used in this study

hmox1a ISH fwd	5’ CATTAACCCTCACTAAAGGGAACAGGACTTGGAGCACTTCTTCGG 3’
hmox1a ISH rev	5’ TAATACGACTCACTATAGGGAGACAACTCAAAGCGTTTATGGACAG 3’
ctsl.1 ISH fwd	5’ CATTAACCCTCACTAAAGGGAAGATCAGAAACAGTGTGGATCATGC 3’
ctsl.1 ISH rev	5’ TAATACGACTCACTATAGGGCCTAGACAGGGGCATTAAAAATGAAAACAG 3’
slc40a1 ISH fwd	5’ CATTAACCCTCACTAAAGGGAAAAAACCTGTCGCCGAAGTTCAC 3’
slc40a1 ISH rev	5’ TAATACGACTCACTATAGGGATAATCCTCCCACTGCGATGG 3’
calr fwd	5’ CACCATGACTGCGTTATCCCTACTGTTTATG 3’
mTagBFP fwd	5’ CACCATGAGCGAGCTGATTAAGGAGAACA 3’
mTagBFP rev	5’ TTAATTAAGCTTGTGCCCCAGTTTGCT 3’
*calr*NOKDEL NO STOP rev	5’ GAGTTTAGAGTCTGTTTCCTCCTCCTC 3’
calr3a fwd	5’ CACCATGCGGATCACTGCTGCA 3’
calr3a rescue fwd	5’ CACCATGAGAATAACAGCCGCCGTGTGCTTTATTTCTGCACTGGC 3’
calr3a rev	5’ GATCATCTAGACTACAATTCATCTTTAGGGAGCGCATCAT 3’
*calr3a*NOKDEL NO STOP rev	5’ AGGGAGCGCATCATCCTCA 3’
calr3b fwd	5’ CACCATGCAAATTTCATTATTACAGTTAATTTCGGCT 3’
calr3b rev	5’ GATCATCTAGACTACAGTTCGTCTTTCTGAAGCACATC 3’
*calr3b*NOKDEL NO STOP rev	5’ CTGAAGCACATCCTCGTCTCC 3’
