# Supplementary material for: Quality assurance of hematopoietic stem cells by macrophages determines stem cell clonality
Source: Science. Author manuscript; Available in PMC 2022 Sep 30. (PMC9524573; doi:10.1126/science.abo4837)
Supplement: Table S2 [file NIHMS1835312-supplement-Table_S2.pdf]

Table S2. Sequences for morpholinos used in this study

Morpholino	Sequence	Source
standard control	5' CCTCTTACCTCAGTTACAATTTATA 3'	GeneTools
irf8	5' TCAGTCTGCGACCGCCCGAGTTCAT 3'	(*16*)
calr	5' AACAGTAGGGATAACGCAGTCATCT 3'	This paper
calr3a	5' GCAGCAGTGATCCGCATCTCTGCAC 3'	This paper
calr3b	5' AAAGAAATGACTCCCGCACGCTCGC 3'	This paper
perk	5' ACTGAAACCCCCTTTCCATTGGGAC 3'	(*55*)
ern1	5' CGCCATCACGCACACATATAAACG 3'	This paper
atf6	5' ACATTAAATTCGACGACATTGTGCC 3'	(*56*)
il1b	5' CCCACAAACTGCAAAATATCAGCTT 3'	(*31*)
