# Supplementary material for: Quality assurance of hematopoietic stem cells by macrophages determines stem cell clonality
Source: Science. Author manuscript; Available in PMC 2022 Sep 30. (PMC9524573; doi:10.1126/science.abo4837)
Supplement: Supplement [file NIHMS1835312-supplement-Supplement.pdf]

Science
AAAS

Supplementary Materials for

Quality assurance of hematopoietic stem cells by macrophages determines stem cell clonality

Samuel J. Wattrus[1,2], Mackenzie L. Smith[1,2], Cecilia Pessoa Rodrigues[1,2], Elliott J. Hagedorn[1,2,†], Ji Wook Kim[1,2], Bogdan Budnik[3], Leonard I. Zon[1,2*]

Correspondence to: zon@enders.tch.harvard.edu

This PDF file includes:

Materials and Methods
Figs. S1 to S5
Tables S1 to S2
Captions for Movies S1 to S3

Other Supplementary Materials for this manuscript include the following:

Movies S1 to S3

Materials and Methods

Animal models

Wild-type zebrafish AB, *casper* or *casper*-EKK, and transgenic lines *cd41*:*GFP* (*35*), *runx1+23:EGFP* (*4*), *runx1+23*:*mCherry* [*runx1+23*:*NLS-mCherry*] (*4*), c*myb:GFP* (*36*), *kdrl*:*mCherry* [*kdrl*:*Hsa.hras-mCherry*] (*37*), *Zebrabow-M* (*15*) *draculin:CreER*^{T2} (*38*), *mpeg1:EGFP* (*11*), *mpeg1:mCherry* (*11*), *mpeg1:BFP* [*mpeg1:TagBFP*], *LCR:EGFP* (*39*), *EF1a:mAG-zGem(1/100))*^{rw0410h} (*12*), and *hsp70l:il1b* (*29*) were used in this study. Alternative gene names are listed in parenthesis and full transgene names are listed in brackets. Wildtype C57BL/6J mice (Jackson Labs stock #000664) were also used in this study. All animals were housed at Boston Children's Hospital and handled according to approved Institutional Animal Care and Use Committee (IACUC) of Boston Children's Hospital protocols 20-10-4254R and 00001366.

Single-cell mRNA-seq preparation

For single-cell mRNA-sequencing of embryonic macrophages, several hundred 72 hpf *runx1+23:mCherry;mpeg1:EGFP* embryos were anesthetized with tricaine overdose and transected with a scalpel to harvest tail tissue. For analysis of HSPCs from *irf8* morpholino-injected animals, 72 hpf *runx1+23:mCherry* embryos were anesthetized with tricaine and chopped with razor blades. Tissue was dissociated using Liberase (Roche) and cells were harvested by FACS using a BD FACSAria II (BD Biosciences). We used 3nM DRAQ7 (Abcam) for Live/Dead staining. Viable single cells were FACS sorted into 384-well plates, called cell capture plates, that were ordered from Single Cell Discoveries, a single-cell sequencing service provider based in the Netherlands. Each well of a cell capture plate contains a small 50 nl droplet

of barcoded primers and 10 µl of mineral oil (Sigma M8410). After sorting, plates were immediately spun and placed on dry ice. Plates were stored at -80° C.

Plates were shipped on dry ice to Single Cell Discoveries, where single-cell mRNA sequencing was performed according to an adapted version of the SORT-seq protocol (*40*) with primers described in (*41*). Cells were heat-lysed at 65° C followed by cDNA synthesis. After second-strand cDNA synthesis, all the barcoded material from one plate was pooled into one library and amplified using *in vitro* transcription. Following amplification, library preparation was done following the CEL-Seq2 protocol (*42*) to prepare a cDNA library for sequencing using TruSeq small RNA primers (Illumina). The DNA library was paired-end sequenced on an Illumina Nextseq™ 500, high output, with a 1×75 bp Illumina kit (read 1: 26 cycles, index read: 6 cycles, read 2: 60 cycles)

Single-cell mRNA-seq analysis

For each dataset, paired-end reads were aligned to the zebrafish transcriptome using BWA (*43*). Data was demultiplexed as described in (*44*). Mapping and generation of count tables were automated using the MapAndGo script. We corrected read counts for UMI barcodes by removing duplicate reads that had identical library, cellular, and molecular barcodes and mapped to the same gene. Transcript counts were adjusted using Poissonian counting statistics to yield the number of UMIs detected per cell. Counts were imported into R using the Seurat suite version 3.0 (*45*). Low quality cells were filtered out by keeping only cellular barcodes with greater than 500 UMIs per cell, greater than 500 genes per cell, and less than 9 percent of reads mapping to mitochondrial genes. Data were log normalized and scaled, and the 2000 most variable genes

were identified. We reduced dimensionality using PCA, selecting 15 PCs that explained the majority of variation in the data. PC loadings were used as input for a graph-based approach to cluster cells by type and as input for Uniform Manifold Approximation and Projection (UMAP). Differentially expressed genes were identified using a Wilcox Rank Sum test to compare count data between clusters. Significant genes were obtained using an FDR threshold of 0.05. To combine single-cell transcriptional profiles with FACS profiles, individually sorted cell indices were merged with single-cell mRNA data by matching well ID.

For further analysis of purified *runx1+23:mCherry*$^{+}$ cells, we discarded the reads mapping to ERCC spike-ins and cells with transcripts correlating to mitochondrial and ribosomal genes with a Pearson's correlation coefficient > 0.65. Next, RaceID3 analysis (*46*) was initiated with mintotal =3000, minexp =5, minnumber=5, and internal batch effect using the plates ID as input. For the outlier identification we used probthr =0.001 and outlog=2. Then, the transcription distance was calculated using the compdist() function, followed by clustexp() using the following parameters: clustnr= 30, bootnr =50, samp=1000, metric = pearson, sat= TRUE, FUNcluster = "hclust" to identified the initial number of clusters present in our dataset. We used the plotjaccard() function to identify the saturation point and re-calculated the clustexp() function using cln=8 and sat=FALSE. After RaceID3 benchmarking, we performed VarID analysis (*47*) to infer the local neighborhoods of cells in the same. To determine the k-nearest neighbors for each cell in gene expression space and test if the links are supported by a negative binomial joint distribution of gene expression we applied the pruneKnn function with the following parameters: d= gene expression values from RaceID3 dataset, pcaComp=100, regNB=TRUE, batch=plate ID name, knn=10, alpha =1, ngenes=2000 and links with probabilities lower than pvalue < 0.01

were removed. To visualize the VarID analysis, we performed Louvain clustering and UMAP reduction using the compumap() function. To increase our HSPC cluster resolution, we removed the erythrocytes and myeloid cells clusters from our single-cell object using the reduceset() function. Differentially expressed genes were identified by the differential gene expression function diffexpnb() from the RaceID3 (v0.1.4) algorithm with multiple testing corrected by the Benjamini-Hochberg method set significance as $p<0.01$. ClurterProfiler software version 4.0 (https://doi.org/10.1016/j.xinn.2021.100141) was run to evaluate the biological pathways associated with each of differentially expressed genes (DEG) from the VarID identified clusters. The parameters applied were: all identified transcript set as universe, the DEG form each target cluster set as gene target list and enrichment strategy set either as enrichGO() or enrichKEGG(). Significance was scored by adjusted Benjamin-Hochberg with p-value = 0.01 and qvalue = 0.05.

Whole mount in situ hybridization (WISH)

In situ hybridization was performed using a standard protocol (*48*). *In situ* probes were generated by PCR amplification using a cDNA or plasmid template followed by reverse transcription with digoxigenin-linked nucleotides. Primer sequences for all WISH probes used in this paper are provided in Supplementary Table 1.

Transgenesis

Transgenic lines were established as previously described (*49*). The *mpeg1:TagBFP* line was generated by PCR amplification off the pCAG-dGBP1-TagBFP plasmid, D-TOPO cloning into pME Gateway, and LR Gateway Reaction to generate constructs with the *mpeg1* promoter driving *TagBFP*, followed by an SV40 polyA signal sequence, all flanked by Tol2 integration

sites. The pCAG-dGBP1-TagBFP plasmid was a gift from Connie Cepko (*50*) (RRID:Addgene_80086). Calreticulin overexpression constructs were generated by PCR amplification off of genomic DNA, followed by D-TOPO cloning and LR gateway reactions as noted before. The fidelity of all constructs was confirmed by sequencing prior to injection.

Microscopy and image analysis

Time-lapse microscopy was performed using a Yokogawa CSU-X1 spinning disk mounted on an inverted Nikon Eclipse Ti microscope equipped with dual Andor iXon EMCCD cameras and a climate controlled (maintained at 28.5˚C) motorized x-y stage to facilitate tiling and imaging of multiple specimens simultaneously. Screening of injected constructs and imaging of WISH embryos was performed using a Nikon SMZ18 stereomicroscope equipped with a Nikon DS-Ri2 camera. Animals were only included for imaging and analysis if expression of all transgenes could be identified. All images were acquired using NIS-Elements (Nikon), blinded, and processed using Imaris (Bitplane). Specimens were mounted in 0.8% LMP agarose with tricaine (0.16 mg/ml) in glass bottom 6-well plates and covered with E3 media containing tricaine (0.16 mg/ml).

Flow cytometry

To collect kidney marrows, adult zebrafish (2 to 9-months-old) were anaesthetized with 0.02% tricaine in fish water and dissected under a Leica MZ75 light microscope. The soft tissue of the kidney marrow was placed in cold 0.9x DPBS (Gibco) with 2% fetal bovine serum (FBS, Gemini Bio-Products) and 1 USP units/mL heparin (Sigma), and then mechanically dissociated by repeated pipetting into single-cell suspension, and passed through a 40-µm nylon mesh 5-10

minutes prior to analysis. To collect embryonic samples, embryos were chopped with a razor blade in cold PBS and then incubated in Liberase (Roche) for 20 minutes at 37˚C before filtering the dissociated cells through a 40 μm filter and transferring to 2% FBS. For samples stained for Calreticulin, Liberase was not added. Cells were blocked in 5% Normal Goat Serum + 0.5% Sodium Azide in cold PBS for 20 minutes, washed, and incubated with a Chicken anti-Calreticulin antibody (abcam ab94935, 1:1000, RRID:AB_10675983) for 30 minutes on ice. Samples were then washed before incubation with a Goat anti-Chicken Alexa Fluor 488 antibody (Invitrogen A11039, 1:1000) for 25 minutes at room temperature, protected from light. CellROX Deep Red (Invitrogen C10422) and Annexin V-FITC (BD Biosciences) staining was performed according to manufacturer instructions. We used 3nM DRAQ-7 for live/dead stain (Abcam). Flow cytometric analysis was performed on a BD FACSAria II with special order 445nm laser for CFP detection when using Zebrabow (BD Biosciences). Gates were drawn using negative and isotype matching controls. Data were analyzed with FlowJo software version 10.

Zebrabow color labeling

At 24 hours post-fertilization (hpf), embryos were transferred to 6-well plates at a density of 25-35 embryos per well and treated with 15 μM 4-hydroxytamoxifen (4-OHT) for 3-5 hours in the dark at 28.5°C. Embryos with dim transgene expression were excluded and the only the brightest 25-35 embryos per condition were included for analysis to account for variation in Zebrabow transgene insert number.

Zebrabow analysis

Color barcodes from Zebrabow kidney marrow samples were quantified using previously published pipelines (*7*) adapted to a Python-based interface. The granulocytic color output was chosen as a read out of clonal changes, as these cells have a short half-life and reflect changes in the HSPC clonal output in a timely manner. Only zebrafish with greater than 75% recombination efficiency were processed. All samples were blinded prior to analysis and compared against clutch-matched siblings.

Drug treatment

Drugs were added to embryo E3 media in 6-well plates with 20-30 embryos per well at 48 hpf and incubated for 24 hours. MEK1/2 inhibition was done using PD98059 (Sigma) at a concentration of 15 μM. Diphenylene iodonium (Sigma) was added to embryos at a concentration of 100 μM. L-Glucose and D-Glucose (Sigma) were added to embryos at 1% weight/volume. Hydrogen peroxide (Sigma) was added to embryos at 2 mM.

Morpholino injections

Morpholinos (GeneTools) were resuspended to 300 μM in nuclease free water, heated to 65°C for 5 minutes, and kept at room temperature. Embryos were injected into the yolk at the 1-4 cell stage with 1-8 ng of morpholino. Morpholino sequences are listed in Supplemental Table 2.

Liposome injection

Zebrafish embryos were dechorionated and anesthetized with tricaine (0.16 mg/ml) on flat agarose disks. Approximately 1.5 nanoliters of liposomes loaded with either clodronate or PBS

(Liposoma) were injected directly into circulation into either the caudal vein (28 hpf) or the duct of Cuvier (48 hpf, 72 hpf, 96 hpf, 120 hpf).

Few-cell proteomics

Cell pellets were submitted after FACS purification for mass spectrometry analysis. Cell pellets were resolubilized in pure water before undergoing the mPOP procedure (*51*). Briefly, cells frozen in water at -80C were put straight into a heated 96 well plate shaker at 95 C for 5 min. After cells were chilled to room temperature, trypsin was added for a 3 hour digest at 38 C. Digested proteins were labeled with TMT11plex (Tandem Mass Tags) labels (Thermo-Fisher, Germany) according to the previously published procedure SCOPE-MS (*17*). After labeling, all samples were quenched with 5% hydroxyl solution and pulled together as one sample for further mass spectrometry analysis. Two consecutive LC-MS/MS experiments were performed on HFX Orbitrap (Thermo-Fisher, CA) equipped with UltiMate 3000 HPLC Nano tandem pump (Thermo-Fisher, CA). Peptides were separated onto PharmaFluidics (Belgium) trapping column followed by elution into 50 cm PharmaFluidics analytical column. Separation was achieved through applying a gradient from 5–27% ACN in 0.1% formic acid over 180 min at 200 nl/min. Electrospray ionization was enabled through applying a voltage of 2 kV using a home-made electrode junction at the end of the microcapillary column and sprayed from stainless steel 4 cm needles (PepSep, Denmark). The HFX Orbitrap instrument was operated in data-dependent mode for the mass spectrometry methods. The mass spectrometry survey scan was performed in the Orbitrap in the range of 400 –1,400 m/z at a resolution of 1.2 × 105, followed by the selection of the twenty most intense ions (TOP10) for HCD-MS2 fragmentation in the Orbitrap using a precursor isolation width window of 0.7 Th, AGC setting of 50,000, and a maximum ion

accumulation of 200 ms. Singly charged ion species were not subjected to HCD fragmentation. Normalized collision energy was set to 34 V and an activation time of 1 ms. Ions in a 10 ppm m/z window around ions selected for MS2 were excluded from further selection for fragmentation for 90 seconds. Raw data were submitted for analysis in Proteome Discoverer 2.4. (Thermo-Fisher, CA) software. Assignment of MS/MS spectra were performed using the Sequest HT and Byonic v3.5 (Protein Metrics, CA) algorithms by searching the data against a protein sequence database including all entries from Zebrafish database and other known contaminants such as human keratins and common lab contaminants. Sequest HT and Byonic searches were performed using a 10 ppm precursor ion tolerance and requiring each peptides N-/C termini to adhere with Trypsin protease specificity, while allowing up to two missed cleavages. 6-plex TMT tags on peptide N termini and lysine residues (+229.162932 Da) was set as static modifications while methionine oxidation (+15.99492 Da) was set as variable modification and deamidation of Asparagine and Glutamine amino acids. An MS2 spectra assignment false discovery rate (FDR) of 1% on protein level was achieved by applying the target-decoy database search. Filtering was performed using a Percolator (*52*) (64bit version).

For quantification, a 0.02 m/z window centered on the theoretical m/z value of each the six reporter ions and the intensity of the signal closest to the theoretical m/z value was recorded. Reporter ion intensities were exported in result file of Proteome Discoverer 2.4 search engine as an excel tables. The total signal intensity across all peptides quantified was summed for each TMT channel, and all intensity values were adjusted to account for potentially uneven TMT labeling and/or sample handling variance for each labeled channel. All further data evaluation for

statistically differential analysis were performed on in-house made R package based on Bioconductor programs (https://www.bioconductor.org/).

Immunofluorescence Staining

Mouse embryos were harvested at 14.5 days post-conception. Fetal livers were dissected and stored in 4% paraformaldehyde at 4°C overnight. Samples were dehydrated in 30% sucrose for 8 hours and embedded in OCT. Cryosections were collected at a thickness of 10 microns. Slides were washed three times in PBS + 0.1% Triton, blocked with 5% normal goat serum and 1% bovine serum albumin for two hours, and stained overnight with rabbit anti-c-Kit (Cell Signaling Technology #3074, 1:400, RRID:AB_1147633) and rat anti-F4/80 (abcam ab6640, 1:100, RRID:AB_1140040). Slides were washed five times with PBS + 0.1% Triton and stained for two hours with Goat anti-rabbit Alexa Fluor 488 (Invitrogen A11008, 1:500, RRID:AB_143165), Goat anti-rat Alexa Fluor 568 (Invitrogen A11077, 1:500, RRID:AB_141874) and DAPI (abcam ab228549, 1:1000). Slides were washed 5 times with PBS + 0.1% Triton and coverslips were mounted with ProLong™ Diamond Antifade Mountant.

Zebrafish EdU Labeling

Embryonic circulation was injected at 72 hpf with 1 nanoliter of 500μM EdU. Embryos were kept at 4°C for 1 hour, fixed in 4% paraformaldehyde for 1 hour, permeabilized with 0.1% Triton for 20 minutes at room temperature, and labeled with Alexa Fluor 647 using the Click-iT reaction (Thermo Fisher) for 30 minutes according to manufacturer instructions. Embryos were washed with PBS+0.5% Triton and blocked for 1 hour in 10% Normal Goat Serum, 0.5% Bovine Serum Albumin, 0.5% Triton. Samples were incubated in Rat anti-mCherry Alexa Fluor

594 (Invitrogen M11240, 1:200, RRID:AB_2536614) for 1 hour at room temperature and washed 5 times with PBS+0.5% Triton.

Zebrafish Embryo Heat Shock

Approximately 30 embryos were transferred to a 50 ml conical tube in 5 ml E3 media and submerged in a 38 C circulating water bath for 30 minutes. To allow for heat shock induced transcription and translation, embryos were kept for 6 hours post-heat shock before analysis.

Parabiosis

Parabiotic zebrafish were generated as previously described (*53*). Briefly, zebrafish embryos were injected with morpholinos, and incubated until the 1,000-cell stage. Embryos were dechorionated, embedded in 4% methylcellulose, and immersed in a high-calcium Ringer's solution (116 mM NaCl, 2.9 mM KCl, 10mM $CaCl_2$, 5mM HEPES, in E3 media) containing penicillin-streptomycin (50U/ml), ampicillin (50U/ml), and kanamycin (0.5 µg/ml). Pairs were fused by positioning partner embryos in contact with each other at the animal pole and wounding at the embryo margin with a prepared glass needle with a 20-micron diameter. Cells were stitched across between parabiont partners and allowed to adhere before needle removal. Successful fusion was indicated by the formation of an unbroken bridge of cells between embryos and dual gastrulation over the subsequent 24 hours. Parabiotic fusions were screened for shared circulation using brightfield microscopy and mounted for confocal imaging. HSPCs and macrophages from both parabiont partners are able to seed the reciprocal niche.

Acridine Orange Staining

Acridine Orange dye (Invitrogen A3568) was used at a final concentration of 3 μg/mL in E3 embryo media. Embryos were incubated with dye for 30 minutes, washed twice with E3, and mounted for imaging.

Statistical analysis

Graphs and statistical analyses were done with Prism (Graphpad), Excel (Microsoft), and RStudio. For all graphs, error bars indicate mean +/- standard deviation. *P* values were obtained with two-tailed Student's *t*-test or One-way ANOVAs for all analyses as indicated. For comparisons between two sample groups, a two-tailed Student's *t*-test was used. For comparisons between multiple groups, One-way ANOVAs were used. Sample sizes were chosen based on sample availability and power calculations determined from preliminary observations to detect a change of at least 33% with an α of 0.05 and a β of 0.8. For all experiments except Zebrabow color labeling, a randomized set of embryos from a mixture of clutches was split into control and perturbation conditions. For Zebrabow experiments a randomized set of embryos from single clutches were split into control and perturbation conditions. All datapoints plotted throughout represent biological replicates. All experiments except for sequencing and proteomics experiments were repeated at least once.

Fig. S1. Macrophages specifically interact with HSPCs.

(**A**) Quantification of macrophage contacts with *kdrl*$^{+}$ endothelial cells (ECs) and *LCR*$^{+}$ red blood cells (RBCs) in the CHT shows minimal levels of interaction compared to the fraction of *runx1+23*$^{+}$ HSPCs engaged by macrophages. Mean +/- s.d. (**B**)(**C**) The only Acridine Orange^{+} or Annexin V-YFP^{+} apoptotic HSPCs in the CHT are those which have been engulfed by macrophages. At baseline, there are no apoptotic HSPCs. Mean +/- s.d., Unpaired t test; ****P<0.0001. (**D**)(**E**) Cells labeled by *cd41:GFP* and c*myb:GFP* - transgenes which also mark HSPCs – undergo intimate interactions with macrophages in the CHT which involve fragments of cytoplasmic fluorescent material exchanged or full cell engulfment. (**F**) Immunofluorescence staining of murine fetal liver at E14.5 reveals that approximately 33% cKit^{+} hematopoietic progenitors are in contact with F4/80^{+} macrophages, including interactions in which pieces of or entire cKit^{+} cells appear to be taken by macrophages.

Fig. S2. Interacting macrophages are a distinct population.

(**A**) GO-term enrichment (*54*) for genes highly enriched in the macrophages which have taken up fragments of or entire HSPCs. Arrows indicate GO-terms of note: lysosome, cholesterol transport, and phagosome. (**B**) Lysotracker dye marks macrophages which interact with HSPCs in the CHT. Scale bar indicates 30 µm. Mean +/- s.d., Unpaired t test; ****P<0.0001. (**C**) The cholesterol mimic, BODIPY, is specifically taken up by macrophages which interact with HSPCs. Scale bar indicates 30 µm. Mean +/- s.d., Unpaired t test, ****P<0.0001. (**D**) UMAP of single-cell mRNA-seq for 72 hpf niche macrophages shows enrichment for the marker *hmox1a* in the subset of macrophages which interact with HSPCs. Whole mount *in situ* hybridization confirms expression of *hmox1a, ctsl.1,* and *slc40a1* in a subset of macrophages in the CHT at 72 hpf. Spectral scale indicates z score.

Fig. S3. Calreticulin cell-autonomously regulates grooming and dooming.

(**A**)(**B**) The fraction of HSPCs interacting with macrophages decreases after injection of either the *calr3a* or *calr3b* morpholino but can be rescued by co-injection of either mRNA or DNA constructs encoding ubiquitous Calreticulin expression. Mean +/- s.d., One-way ANOVA; *P<0.05, **P<0.01. (**C**) Schematic of parabiosis experiment to assess Calreticulin cell autonomy. Embryos with labeled HSPCs or macrophages are injected with either the *calr3a*, *calr3b*, or control morpholino and surgically fused to generate heterotypic parabionts with shared circulation. HSPCs and macrophages migrate across both partners and facilitate analysis of interactions between Calreticulin knock-down and control cells. (**D**) Example and quantification of parabiotic fusion of *runx1+23:mCherry* and *mpeg1:EGFP* embryos. HSPCs with *calr3a* or *calr3b* knock-down have reduced interactions with control macrophages. Knock-down of *calr3a* or *calr3b* in macrophages has no effect on macrophage-HSPC interactions. Mean +/- s.d., One-way ANOVA with Dunnett’s multiple comparisons test; *P <0.05, **P<0.01. (**E**) Knock-down of *calr3a* or *calr3b* reduces the fraction of HSPCs that are groomed or doomed. Mean +/- s.d., One-way ANOVA; *P<0.05, **P<0.01. (**F**) Most HSPCs overexpressing surface-translocated forms of *calr* (84%), *calr3a* (85%), or *calr3b* (71%) are fully engulfed by macrophages.

Fig. S4. Calreticulin knock-down reduces the number of HSPCs in the CHT.

(**A**) Quantification of *kdrl:mCherry;cd41:GFP*$^+$ cells in the floor of the dorsal aorta from 32-46 hpf reveals no significant change in the number of emerging HSPCs in embryos injected with *calr3a, calr3b,* or *irf8* morpholinos. Mean +/- s.d. (**B**) Serial imaging of *cd41:GFP* embryos identify reduced numbers of HSPCs in the CHT at 3 dpf in the absence of macrophage interactions. Similar numbers of cells emerge from the aorta and initially seed the CHT, but embryos injected with the *irf8, calr3a,* or *calr3b* morpholinos exhibit a deficit from 60 – 84 hpf. Mean +/- s.d. (**C**) Quantitation of *cd41:GFP*$^+$ cells in the CHT at 48 hpf, 57 hpf, and 72 hpf. Mean +/- s.d., Unpaired t test; *P<0.05, ***P<0.001. (**D**) Annexin V staining and flow cytometry after injection with either the *calr3a, calr3b,* or *irf8* morpholino finds similar fractions of *runx1+23*$^+$ cells undergoing cell death compared to control morphants. (**E**) Examples and quantitation of *cmyb in situ* hybridization to visualize colonization of the adult marrow in *calr3a* and *calr3b* morphants. Arrows indicate *cmyb* signal in the kidney marrow. Knock-down of *calr3a* or *calr3b* does not alter colonization of the adult marrow from 4 to 6 dpf.

Fig. S5. Macrophage-derived *il1b* drives HSPC cycling through ERK/MAPK.

(**A**) Increasing ROS by treatment with hydrogen peroxide or D-Glucose, which has previously been shown to increase HSPC ROS (*26*), increases the frequency of macrophage-HSPC interactions compared to the non-metabolically active L-Glucose. Mean +/- s.d., Unpaired t test; *P<0.05, **P<0.01. (**B**) Morpholino knock-down of the specific ER stress mediator *perk* reduces macrophage-HSPC interactions, in agreement with prior studies identifying PERK-dependent ER stress as a critical determinant of surface Calreticulin exposure, distinct from IRE1 (*ern1*) or ATF6 (*atf6*) mediated ER stress (*27*). Mean +/- s.d., Unpaired t test; *P<0.05. (**C**) Both macrophage depletion and MAPK inhibition reduce HSPC proliferation. The combination of macrophage depletion and MAPK inhibition has no additional impact on HSPC divisions. Mean +/- s.d., Unpaired t test; *P<0.05, **P<0.01, ***P<0.001. (**D**) Macrophages in the CHT express *il1b*, which has been previously shown to induce HSPC division (*29*, *30*). Spectral scales report z score. (**E**) Schematic of parabiosis experiment to assess *il1b* in macrophage associated divisions. Embryos with $mCherry^+$ HSPCs (*runx1+23*$^+$) and $EGFP^+$ macrophages (*mpeg1*$^+$) are injected with a control morpholino and surgically fused to embryos with BFP^+ macrophages (*mpeg1*$^+$) injected with an *il1b* morpholino. The frequency of HSPC division after macrophage interaction is evaluated for control ($EGFP^+$) and *il1b* knock-down macrophages (BFP^+) within the same animal. (**F**) Example and quantification of parabiotic fusion of *runx1+23:mCherry;mpeg1:EGFP* control to *mpeg1:BFP il1b* knock-down embryos. Knock-down of *il1b* in macrophages significantly reduces the frequency of associated HSPC divisions. Mean +/- s.d., Paired t test; *P<0.05. (**G**) Treatment with an ERK/MAPK inhibitor abrogates the pro-proliferative effect of *il1b* stimulation. Mean +/- s.d., One-way ANOVA with Sidak's multiple comparisons test. **P<0.01.

Movie S1. Macrophages take up fluorescent material from HSPCs.

Video of Fig. 1A; an *mpeg1:mCherry*$^{+}$ crawls over the surface of a *runx1+23:EGFP*$^{+}$ HSPC at 72 hpf and sucks up cytoplasmic EGFP material.

Movie S2. Macrophages may groom or doom HSPCs in the CHT niche.

Video showing *mpeg1:EGFP* macrophages interacting with *runx1+23:mCherry* HSPCs in the CHT at 72 hpf. Arrows indicate fully engulfed "doomed" HSPC that is degraded, and an HSPC that is "groomed" and has fragment of material taken up. Circle indicates HSPC about to divide.

Movie S3. HSPCs only undergo apoptosis upon dooming.

Video showing a *runx1+23:mCherry*$^{+}$ HSPC being doomed by an *mpeg1:BFP*$^{+}$ macrophage in the CHT at 72 hpf. The cell does not become Acridine Orange^{+} until it is fully engulfed.
